# Post-tuberculosis health-related quality of life, lung function and exercise capacity in a cured pulmonary tuberculosis population in the Breede Valley District, South Africa

**DOI:** 10.4102/sajp.v75i1.1319

**Published:** 2019-07-31

**Authors:** Kurt J. Daniels, Elvis Irusen, Hamilton Pharaoh, Susan Hanekom

**Affiliations:** 1 Division of Physiotherapy, School of Health Sciences, University of KwaZulu-Natal, Durban, South Africa; 2 Department of Internal Medicine, Faculty of Health Science, Stellenbosch University, Cape Town, South Africa; 3 Division of Physiotherapy, Faculty of Health Sciences, Stellenbosch University, Cape Town, South Africa

**Keywords:** Pulmonary Tuberculosis, Lung Function, Health-Related Quality of Life, Exercise Capacity, Rural Health

## Abstract

**Background:**

Pulmonary tuberculosis (PTB) remains a major concern worldwide. Albeit curable, PTB continues to negatively affect patients’ health-related quality of life (HRQoL) and functioning even after cure.

**Objectives:**

To describe the demographics, respiratory symptoms, pulmonary airflow patterns, HRQoL and exercise capacity of cured PTB patients, in the Breede Valley district of South Africa.

**Methods:**

A cross-sectional study conducted at five primary health care facilities included adult patients diagnosed with PTB, who had completed anti-tuberculosis treatment. Post-treatment bronchodilator lung function, HRQoL and 6-min walk distance (6MWD) were measured.

**Results:**

Three hundred and twenty-four patients were screened. Specific challenges resulted in 45 patients being included (male *n* = 25 [56%]; mean population age 39.9 [± 10.2]). HRQoL was assessed using the short-form 12v2, part of the burden of lung disease core questionnaire. In general, self-reported physical scores (physical health component summary score = 45) were higher than mental scores (mental health component summary score = 39). The mean 6MWD was 294.5 m (± 122.7) m (range 110 m – 600 m), which is well below normal reference values. Forty-eight percent (48%) of the sample presented with abnormal lung function, including obstructive (*n* = 9; 21%), restrictive (*n* = 11; 25%) and mixed (*n* = 1; 2%).

**Conclusions:**

This pilot study suggests that most cured PTB patients have decreased HRQoL, exercise capacity and abnormal lung function. This study is the first to describe the combination of these three outcomes in a South African population.

**Clinical implications:**

Clinicians must recognise that holistic management of PTB patients is required after cure.

## Introduction

Traditionally, medical outcomes were measured using objective clinical indicators such as physiological tests and disease status. Recently, the need has arisen to assess health status beyond the traditional indicators of morbidity and mortality (Chamla 2004). This evolution in the philosophy of modern medicine has led to the inclusion of patients’ perspective and quality of life in the evaluation of medical outcomes (Santana 2008).

Global strategies to combat pulmonary tuberculosis (PTB) are focussed on microbiologic markers and outcomes such as cure, mortality, and treatment completed or failure (Aggarwal et al. 2013). However, recent literature has highlighted concerns relating to the impact of the disease on patients’ health (World Health Organization 2015b). According to the World Health Organization’s definition of health, ‘a state of complete physical, mental and social well-being and not only the absence of disease or infirmity’ (World Health Organization 1964:1), current strategies to holistically eradicate tuberculosis are inadequate. This strategic shortfall could have economic implications for middle-lower income countries as 90% of new smear-positive cases fall within the economically active age group (English 2009). Therefore, a greater understanding of how the disease affects health-related quality of life (HRQoL), functional capacity and pulmonary function could inform the development of more holistic management strategies.

Pulmonary tuberculosis has substantial adverse impacts on patients’ quality of life. Patients’ perceived HRQoL is decreased in all patients diagnosed with PTB. While HRQoL improves as pharmacological treatment progresses (Marra Marra et al. 2008), perceptions of both mental and physical quality of life remain below the population normal (Aggarwal et al. 2013). Some would argue that further improvements in HRQoL could naturally occur over time in the absence of active disease. However, in a population of multi-drug resistant (MDR) TB sufferers, the decreased perception of HRQoL was still prevalent 18 months after the patients were deemed cured of TB (Hnizdo, Singh & Churchyard 2000).

Exercise capacity in PTB populations, predominantly ascertained via the 6-min walk test (6MWT), has been assessed in a number of studies (Ando et al. 2003; Castanho et al. 2012; Pontororing et al. 2010; Sivaranjini, Vanamail & Eason 2010; Yoshida et al. 2006). The impact of PTB on walking distance varies depending on age and the severity of the disease. Studies investigating the change in exercise capacity during active treatment have reported improvements when compared to baseline. However, in cross-sectional studies, when compared to a normal population, the 6MWT distance (6MWD) is significantly reduced in PTB patients, even in those patients who had successfully completed their pharmacological treatment regimen.

The association between PTB and lung function impairment has been understood for several years (Snider et al. 1971). Pulmonary tuberculosis has recently been identified as an independent risk factor for the development of chronic obstructive pulmonary disease (COPD), in major population-based studies such as the Pravalencia de EPOC en Columbia (PREPCOL) and Proyecto Latinomericano de Investigación Obstrucción Pulmanar (PLATINO) studies (Menezes et al. 2007). Consequences of PTB include permanent scarring, bronchiectasis and pleural fibrosis (Chakaya, Kirenga & Getahun 2016).

During the treatment of active PTB, lung function impairment is usually restrictive in nature. This may persist or develop into an obstructive pattern (Chakaya et al. 2016). A recent systematic review estimated that patients older than 40 years of age are three times more likely to develop COPD when they have a history of PTB (Byrne et al. 2015). Albeit that PTB is a risk factor for COPD, the spirometric values are often influenced by concurrent risk factor exposure, such as smoking, biomass fuel exposure, dust and childhood respiratory illness, making it difficult to distinguish pure obstructive abnormalities from other lung structural abnormalities without full body plethysmography (Plit et al. 1998). However, a recent South African study has identified that patients diagnosed with PTB do suffer from lung function abnormalities (Manie et al. 2016).

It is evident that PTB has a continued effect on HRQoL, exercise capacity and pulmonary function that can be regarded as sequelae of the disease. The clinical burden of the disease may, therefore, extend beyond microbiological cure. The global success of TB intervention strategies has given rise to an increasing number of TB survivors living with the sequelae of the disease (Chushkin & Ots 2017). Despite reports of all three components in previous studies, no study has reported on the simultaneous effects of all three components in a single post-PTB population.

As we move beyond the Stop TB strategy (World Health Organization 2006) and towards the End TB strategy (World Health Organization 2015a), a greater understanding of the latent effects of PTB sequelae is needed. This understanding could inform the development of a targeted rehabilitation intervention strategy for the cured PTB population. The aim of this study was to describe the demographics, respiratory symptoms, pulmonary airflow patterns, HRQoL and exercise capacity of PTB patients who have successfully completed treatment, in the Breede Valley district of South Africa.

## Methods

This article was adapted from the thesis work of Daniels (2015). We conducted this study in the Breede Valley, a predominantly rural region about 112 km from Cape Town central business district. We identified five primary health care facilities (PHCFs) as sites and these were available for patient recruitment.

This cross-sectional study employed a sample of convenience. Adult patients (18 years and older), diagnosed with PTB and successfully followed up through the course of their pharmacological treatment by the Cape Winelands District health care system, were considered for the study. Patients were included if they had at least two negative sputum sample results and had completed at least 5 months of pharmacological PTB treatment. Patients were excluded if they were not contactable, presented with haemoptysis, pneumothorax, and unstable cardiovascular status, recent abdominal or thoracic surgery or did not provide consent.

Prior to the commencement of data collection, the first author was trained in the spirometry manoeuvre based on the American Thoracic Society (ATS) guidelines (ATS 2005) by qualified lung function technicians at a tertiary hospital. The research assistant (RA) was trained by the first author for the collection of height and weight data as well as the execution of the 6MWT according to ATS guidelines (ATS 2002).

### Health-related quality of life (the burden of lung disease core questionnaire)

The burden of lung disease (BOLD) core questionnaire (Buist, Vollmer & McBurnie 2008) has been validated in several national and international studies, following a standardised methodology. The questionnaire has been translated into Afrikaans and tested in one sample within the Cape Metropole (Buist et al. 2008). The questionnaire also incorporates the short-form 12v2 (SF-12v2) questionnaire, a shortened form of the SF-36v2, which is used to assess patient’s HRQoL. The SF-12v2 shows good correlation to the SF-36v2 (Ware et al. 1996).

The 12 items reflect the following eight sub-domains: self-perceived general health (GH), bodily pain (BP), physical functioning (PF), role physical (RP), vitality (VT), role emotional (RE), mental health (MH) and social functioning (SF). A physical health component summary score (PCS) and a mental health component summary score (MCS) were generated for each patient using the Quality Metric software scoring algorithm.

The scores were then normalised to be comparable with a mean population score of 50. The lower the PCS or MCS, the more activity limitation the person has. The questionnaire includes information on respiratory symptoms (cough, sputum, wheezing and shortness of breath), occupation, respiratory diagnosis (e.g. asthma, emphysema, COPD, chronic bronchitis and TB), co-morbidities, health care utilisation, medication use, activity limitation and health status. We obtained permission to use the questionnaire from BOLD (Buist et al. 2008).

### Exercise capacity – The 6-min walk test

The 6MWT is a valid, reliable, safe and cost-effective test to measure exercise capacity. Several studies have used the 6MWT in a population with TB (Ando et al. 2003; Castanho et al. 2012; Pontororing et al. 2010). The data collection was according to the standardised ATS guideline.

### Lung function

Post-bronchodilator (Buist et al. 2008) spirometry was performed using the Spirobank II (MIR, Roma, Italy) and analysed using the Win Spiro v4.4 software (Andersen et al. 2011; Degryse et al. 2012; Mohammad et al. 2013). The European Respiratory Society ECCS (Economic Community for Coal and Steel) normal reference values were applied. The Spirobank II meets ATS recommendations for accuracy and precision for measuring both forced expiratory volume in 1 s (FEV_1_) and forced vital capacity (FVC) (Liistro Vanwelde et al. 2006) and was found to be valid when compared to the Jaeger Master Scope laboratory spirometer. For this study, calibration was checked at regular intervals using a 3*l* calibration pump and the deviation was not more than 5% as per the manufacturer’s recommendation.

### Data collection and analysis

Data were collected between December 2012 and August 2013. The first author obtained patient names and contact details from the TB registers at the five clinics. Appointments were then scheduled at a clinic for data collection. If patients were unable to attend the clinic, the research team set up appointments for data collection at their homes.

The first author using field spirometry collected lung function data. The spirometry manoeuvre was explained and fully demonstrated by the first author prior to execution by the patient. A minimum of three acceptable manoeuvres was performed (reproducible within 150 mL). The best attempt was recorded and used for analysis. Patients were given 2 min rest between manoeuvres or until their breathing rate returned to normal. Spirometry reports were checked post hoc by a pulmonologist to ensure acceptability and reproducibility.

The 6MWT data were collected by the RA. Patients walked a standardised course, which was marked off by two cones. The standardised 6MWT allows for a 30-m track; however, because of space limitations within the community, distances were standardised to 20 m at the clinics and further reduced to 10 m at patients’ homes. Because of indoor space limitations, all the 6MWTs were conducted outside, on a flat, smooth surface and in favourable environmental conditions. In the primary care setting, this is often not possible and space limitations force clinicians and researchers to modify the course length (Beekman 2013). Beekman (2013) evaluated the impact of a course length of 30 m versus a course length of 10 m on 6MWD in COPD patients and found that a 10 m course significantly shortens the 6MWD that people with COPD achieve (Beekman 2013) when compared to norm values (Casanova et al. 2011). Baseline and post-test heart rate (HR) and oxygen saturation percentage (SPO_2_%) were measured using the Nellcor Oximax N-65 portable pulse oximeter (Covidien, Jersey City, NJ, USA). Patients’ dyspnoea and fatigue were measured before and after the execution of the test using the modified Borg scale.

**FIGURE 1:**
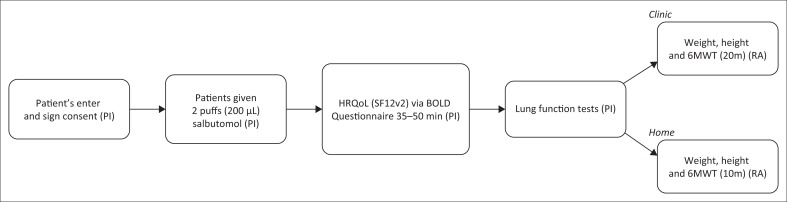
Procedure for data collection at clinics and patient homes.

The primary investigator (PI) collected the HRQoL data using the BOLD core questionnaire in a private location (Figure 1).

All data were entered electronically by the first author and stored on a password-protected laptop. Spirometry data were downloaded to a research laptop and patient information was coded.

### Statistical analysis

**FIGURE 2:**
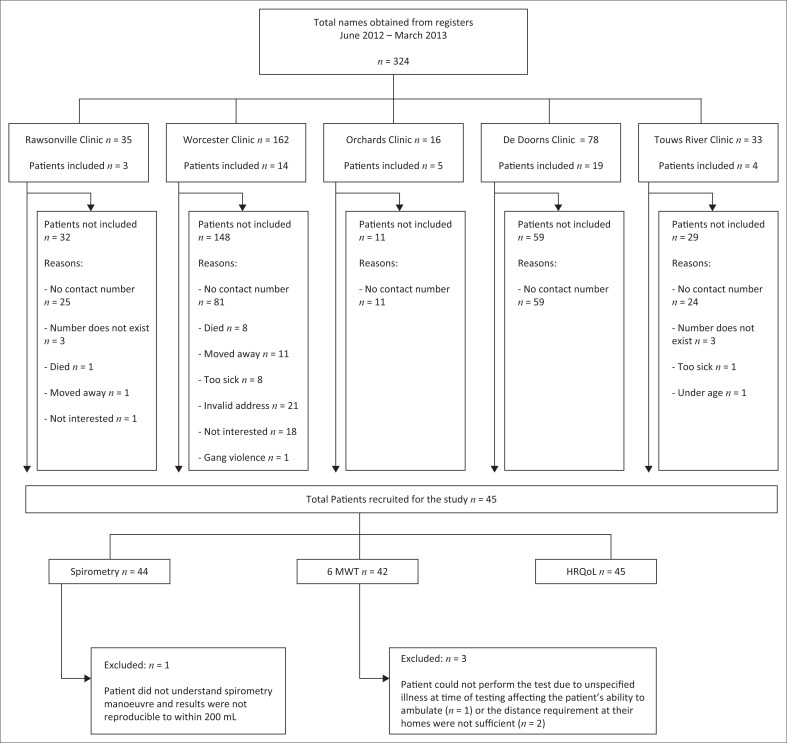
Flow diagram of patients included and excluded from the study.

Data were analysed using Statistica v11. Descriptive statistics were used to describe the basic features of the data. Means and standard deviations were reported for normally distributed data. When data were skewed, medians and interquartile ranges were reported. *T*-test analysis was used to compare the distances of patients who walked the 20 m course versus those who walked the 10 m course. A *p*-value of < 0.05 was considered to be statistically significant. Short-form 12v2 data were analysed using Quality Metric Health Outcomes Scoring Software v4.5. Patients’ data were removed from analysis if they were unable to successfully complete a methodological outcome according to the study criteria or stipulated guidelines (Figure 2).

### Ethical considerations

Ethical approval for the study was obtained from the Committee for Human Research at Stellenbosch University (S12/06/186). Permission was obtained from the District Department of Health and all patients provided written informed consent prior to participating in the study. All patients who participated in the study provided written informed consent.

## Results

A total of 324 patients’ names were obtained from the folders at the five included clinics. Of these, 45 patients were successfully recruited for the study (Figure 2). The majority of patients (*n* = 206; 63%) were not contactable for a variety of reasons (Figure 2). It is difficult to recruit patients for studies in regions like this which has migrant labourers who live in informal housing. Descriptive characteristics of the 45 included in our sample are shown in Table 1.

**TABLE 1:** Descriptive characteristics of population (***n* = 45**).

Variables	%	*n*	Mean ± s.d
**Demographics**			
Gender (male)	56.00	25	-
Age (years)	-	-	39.88 ± 10.20
Mixed Race people	93.00	42	-
Black people	7.00	3	-
Weight (kg)	-	-	55.60 ± 11.21
Height (m)	-	-	1.65 ± 0.11
BMI (kg/m^2^)	-	-	20.53 ± 4.05
Years of formal schooling (years)	-	-	7.8 ± 3.52
**Respiratory conditions or symptoms**			
Diagnosed with TB more than once	64.40	29	-
Number of times diagnosed with TB	-	-	1.86 ± 0.63
Diagnosed emphysema	0.00	0	-
Diagnosed Asthma	20.00	9	-
Diagnosed COPD	6.60	3	-
Breathing problems interfered with ADL	35.50	16	-
Usually cough without a cold	64.40	29	-
Usually cough up phlegm	73.30	33	-
Have had wheezing in the last 12 months	62.20	28	-
**Tobacco use**			
Smoking history	78.00	35	-
Cigarette type			
Manufactured	57.14	20	-
Hand Rolled	25.71	9	-
Both	17.14	6	-
**Occupational exposure**			
Worked for longer than 1 year in a dusty job?	78.00	35	-
Worked in a dusty job and cigarette smoker	17.10	6	-
**Co-morbidities**			
Heart disease	2.00	1	-
Hypertension	8.00	4	-
Lung cancer	0.00	0	-
Stroke	6.00	3	-
**Spirometry**			
FEV1 % predicted	-	-	90.96 ± 32.43
FVC % predicted	-	-	83.15 ± 35.54
FEV1/FVC % predicted	-	-	93.71 ± 14.64
Obstruction pattern	21.00	9	-
Restrictive patter	25.00	11	-
Mixed pattern	2.00	1	-
Normal lung function	52.00	23	-

s.d., standard deviation; TB, tuberculosis; ADL, activities of daily living; BMI, body mass index; COPD, chronic obstructive pulmonary disease.

The study sample consisted of 25 (56%) men (mean age 39.9[± 10.2]). The majority of the sample classified themselves as mixed race (*n* = 42; 93%) and their average number of years of formal schooling was 7.8 (± 3.5) years. Almost two-thirds of the population (*n* = 29; 64%) had been diagnosed with PTB more than once and four (8%) had been hospitalised for breathing problems before they were 10 years old. The mean body mass index (BMI) of 20.5 (± 4.7) fell within population norms for the region. Over three-quarters of the population (*n* = 35; 78%) had a smoking history or had been exposed to occupational dust, while only a small proportion (*n* = 6; 17%) were exposed to both. Only 6.6% (*n* = 3) of patients were previously diagnosed with COPD (Table 1).

**FIGURE 3:**
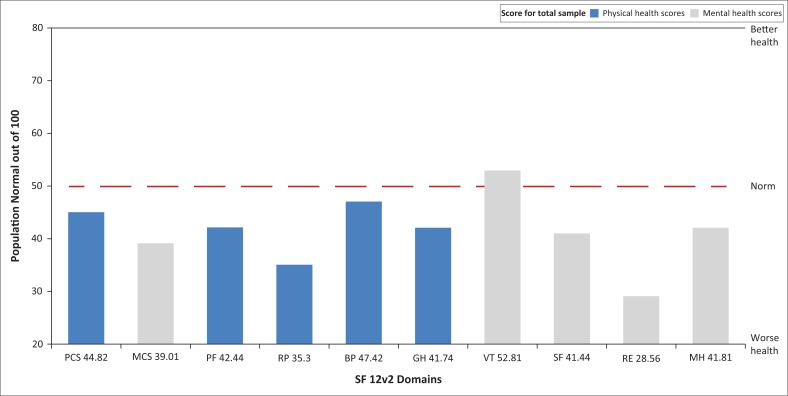
Short-form 12v2 domain scores.

Respondents scored poorly on the majority of sub-domains of the SF-12v2. The mean summary scores obtained for both mental and physical health were lower than the population norm. ‘Role emotional’ and ‘Role physical’ scored lowest with mean scores of 28.1 and 35.3 out of a possible 100, respectively, while vitality scored the highest with 52.8. Both the MCS and the PCS were lower than the population norm. Patients’ perception of their MH was worse than their perception of their physical health (Figure 3).

The mean 6MWD was 294.1 (± 122.7) m (range 110 m – 600 m). The mean walking distance for men and women was 277 m and 317 m, respectively; however, this was not significantly different (*p* = 0.3). While physiological measurements of oxygen saturation (SPO_2_) and HR remained constant from baseline to post-test measurements, patients’ perceptions of dyspnoea (*p* < 0.001) and fatigue (*p* < 0.001) changed from baseline to post-test measurement (Table 2).

**TABLE 2:** Results of the 6-min walk test.

Variable	Baseline	End of test	*p*
SPO_2_* (%)	96.93 ± 3.9	96.95 ± 2.41	0.964
Heart rate* (BPM)	91.06 ± 18.01	92.88 ± 19.19	0.235
Dyspnoea* (modified BORG)	0.79 ± 1.33	1.48 ± 1.64	0.001
Fatigue* (modified BORG)	0.98 ± 1.53	1.70 ± 1.73	0.001

Note: *t*-Test analysis was used to determine the *p*-value of baseline and end of test data.

Key: * = mean ± s.d. and *p*-value; SPO_2_: oxygen saturation.

**FIGURE 4:**
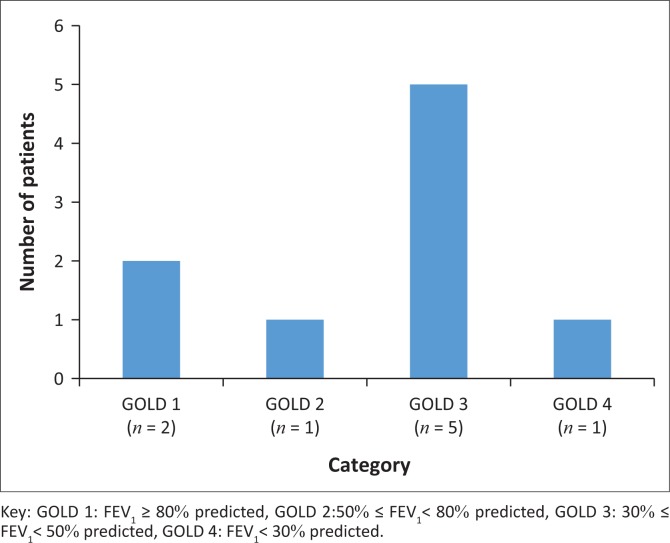
Categorisation of obstructive pattern according to GOLD criteria.

Slightly under half the sample (*n* = 19, 48%) presented with abnormal lung function patterns. Both obstructive and restrictive patterns were observed. An obstructive lung function pattern was observed in 21% (*n* = 9) of the total population. A weak non-significant positive association was present between patients’ FEV_1_ percentage of predicted values and their perception of health (FEV_1_ % predicted vs. MCS; *r* = 0.16; *p* = 0.29) (FEV_1_ % predicted vs. PCS; *r* = 0.29; *p* = 0.59). Exercise capacity showed a significant weak, but slightly more positive association with FEV_1_ percentage of predicted value (FEV_1_ % predicted vs. 6MWD; *r* = 0.31; *p* < 0.001) (Figure 4).

## Discussion

Our findings suggest that patients may suffer from impaired lung function, a decreased exercise capacity and a decreased HRQoL on completion of anti-TB drug therapy. Although all three of these outcomes have been investigated individually at various time points in the management of PTB, to the authors’ knowledge, this is the first study to report on the three outcomes at one time point.

Our results suggest that PTB has a negative impact on patients’ perceived HRQoL in both physical and mental domains. Our study results, therefore, agree with the conclusion of the systematic review by Guo, Marra, and Marra (2009) which concluded that TB has substantial adverse impacts on patients’ quality of life, even after they have been deemed cured (Guo et al. 2009). Both domains will need to be addressed in a holistic management protocol. Whether a link exists between the two domains remains uncertain. Poor perceptions of MH, however, tended to persist well after patients were deemed microbiologically cured.

Our results confirm that patients present with decreased exercise capacity on completion of treatment. The 6MWD of patients in this study was reduced compared to global population normal values; however, they were comparable with the distances covered in a similar South African population (De Grass, Manie & Amosun 2014). The mean 6MWD in the study by De Grass et al. (2014) of the control group at baseline was 340 m (range 160 m – 680 m). The mean distance walked and wide range indicate that urban and rural populations are comparable. This result is similar to the findings of several international studies in the field (Ando et al. 2003; Castanho et al. 2012; Sivaranjini et al. 2010; Yoshida et al. 2006).

The currently available literature is variable about the dominant lung function pattern associated with PTB. This finding is understandable because PTB is known to cause lung tissue scarring (Plit et al. 1998). The damage to the lung and resultant lung function impairment is dependent on the location of the primary PTB lesion (Plit et al. 1998). Whether the lung function impairment will remain ‘stable’ overtime will need to be explored. The degree of lung function impairment does not have a strong positive association with the patients’ perception of their health or their exercise capacity. This further demonstrates that a patients’ perceived health may be worse than the physical parameters observed which then may negatively impact on their quality of life.

## Study limitations

The results of this pilot study should be interpreted with caution for a number of reasons. Firstly, patient recruitment presented a major problem, therefore resulting in a relatively small study sample. Violence and social unrest in the district, lack of transport, lack of patient motivation and understanding, migrant labour and incorrect patient contact details were documented reasons why patients could not be included. Communication with patients was another issue as clinic nurses relied on either community workers or word of mouth to communicate. When patients with valid contact details were unable to attend the clinic, the research team went door-to-door setting up appointments to collect data. Future strategies need to consider these challenges to optimise patient recruitment. Secondly, although the BOLD core questionnaire had been validated in a semi-urban population within the Cape Metropole, it had never been used in a rural population before. The length and complexity of this questionnaire may have influenced patients’ responses, especially in the more rural areas where patients took longer to complete the questionnaire. Finally, adaptations to the 6MWT course were necessary for the rural environment. The 6MWT course length was reduced from 30 m to 20 m at the clinics and 10 m at the patients’ homes because of a lack of space or a lack of uniform surface ground. While our findings were similar to the reported 6MWDs in other PTB populations, normal reference values cannot be assumed for a reduced course length. Researchers should develop suitable reference values for a reduced 6MWD for use in rural settings. The ATS guidelines recommend that the test should ideally be performed indoors in a 30-m straight corridor, and normative values are based on this distance (Beekman 2013).

## Conclusion

The findings of this cross-sectional pilot study suggest that patients who have successfully completed treatment for PTB suffer from impaired lung function, decreased exercise capacity and decreased quality of life. The underlying reasons for these deficits are unknown and require further investigation. Whether these impairments resolve over time is also not clear. What remains clear from these baseline data is that clinicians have to recognise that holistic management of PTB patients is required, even after microbiological cure. Albeit curable, PTB sequelae have not received the same attention as that of COPD; therefore, pulmonary rehabilitation guidelines for post-TB patients do not exist. The data of this pilot study may serve to inform the planning of a prospective, multi-center study with large population controls. This information is critical to understanding TB sequelae and thereby ensuring the return of health of PTB survivors.

## References

[CIT0001] Aggarwal A.N., Gupta D., Janmeja A.K. & Jindal S.K., 2013, ‘Assessment of health-related quality of life in patients with pulmonary tuberculosis under programme conditions’, *International Journal of Tuberculosis and Lung Disease* 17(7), 947–953. 10.5588/ijtld.12.0299 23743314

[CIT0002] American Thoracic Society (ATS), 2002, ‘Guidelines for the six-minute walk test’, *American Journal of Respiratory and Critical Care Medicine* 166(1), 111–117. 10.1164/ajrccm.166.1.at1102 12091180

[CIT0003] American Thoracic Society (ATS), 2005, ‘Standardization of spirometry’, *European Respiratory Journal* 26, 319–338. 10.1183/09031936.05.00034805 16055882

[CIT0004] Andersen A.H., Vinther A., Poulsen L.L. & Mellemgaard A., 2011, ‘Do patients with lung cancer benefit from physical exercise?’, *Acta Oncologica* 50(2), 307–313. 10.3109/0284186x.2010.529461 21231792

[CIT0005] Ando M., Mori A., Esaki H., Shiraki T., Uemura H., Okazawa M. et al., 2003, ‘The effect of pulmonary rehabilitation in patients with post-tuberculosis lung disorder’, *Chest* 123(6), 1988–1995, viewed n.d., from https://www.ncbi.nlm.nih.gov/pubmed/12796179. 12796179 10.1378/chest.123.6.1988

[CIT0006] Beekman E., 2013, ‘Course length of 30m versus 10m has a significant influence on 6-minute walk distance in patients with COPD: An experimental cross over study’, *Journal of Physiotherapy* 59(3), 169–176. 10.1016/S1836-9553(13)70181-4 23896332

[CIT0007] Buist A.S., Vollmer W.M. & McBurnie M.A., 2008, ‘Worldwide burden of COPD in high- and low-income countries. Part I. The burden of obstructive lung disease (BOLD) initiative’, *International Journal of Tuberculosis and Lung Disease* 12(7), 703–708, viewed n.d., from https://www.ncbi.nlm.nih.gov/pubmed/18544191. 18544191

[CIT0008] Byrne A., Marias B., Mitnick C., Lecca L. & Marks G., 2015, ‘Tuberculosis and chronic respiratory disease: A systematic review’, *International Journal of Infectious Diseases* 32, 138–146. 10.1016/j.ijid.2014.12.016 25809770

[CIT0009] Casanova C., Celli B.R., Barria P., Casas A., Casas A., Cote C. et al. 2011, ‘The 6-min walk distance in healthy subjects: Reference standards from seven countries’, *European Respiratory Journal* 37(1), 150–156. 10.1183/09031936.00194909 20525717

[CIT0010] Castanho I.A., Godoy M.D., Menezes S.L., Costa W., Lopes A.J., Pacheco A.G. et al., 2012, ‘The functional assessment of patients with pulmonary multidrug-resistant tuberculosis’, *Respiratory Care* 57(11), 1949–1954. 10.4187/respcare.01532 22417754

[CIT0011] Chakaya J., Kirenga B. & Getahun H., 2016, ‘Long term complications after completion of pulmonary tuberculosis treatment: A quest for a public health approach’, *Journal of Clinical Tuberculosis and Other Mycobacterial Diseases* 3, 10–12. 10.1016/j.jctube.2016.03.001

[CIT0012] Chamla D., 2004, ‘The assessment of patients’ health-related quality of life during tuberculosis treatment in Wuhan, China’, *International Journal of Tuberculosis and Lung Disease* 8(9), 1100–1106, viewed n.d., from https://www.ncbi.nlm.nih.gov/pubmed/15455595. 15455595

[CIT0013] Chushkin M.I. & Ots O.N., 2017, ‘Impaired pulmonary function after treatment for tuberculosis: The end of the disease?’, *Jornal Brasileiro de Pneumologia* 43(1), 38–43. 10.1590/s1806-37562016000000053 28380187 PMC5790675

[CIT0014] Daniels K., Irusen E., Pharaoh H. & Hanekom S., 2015, An investigation into the lung function, health-related quality of life and functional capacity of a cured Pulmonary Tuberculosis population in the Breede Valley, South Africa: A pilot study, viewed 09 May 2019, from https://scholar.sun.ac.za/handle/10019.1/97001. 10.4102/sajp.v75i1.1319 PMC6676936 31392293

[CIT0015] De Grass D., Manie S. & Amosun S., 2014, ‘Effectiveness of a home-based pulmonary rehabilitation programme in pulmonary function and health-related quality of life for patients with pulmonary tuberculosis: A pilot study’, *African Health Sciences* 14(4), 866–872. 10.4314/ahs.v14i4.14 25834495 PMC4370065

[CIT0016] Degryse J., Buffels J., Van Dijck Y., Decramer M. & Nemery B., 2012, ‘Accuracy of office spirometry performed by trained primary-care physicians using the MIR spirobank hand-held spirometer’, *Respiration* 83(6), 543–552. 10.1159/000334907 22269344

[CIT0017] English R., 2009, *Boland/Overberg region. Annual health status report 2007/2008*, Western Cape, viewed 07 July 2019, from https://www.westerncape.gov.za/Text/2009/12/boland_overberg_region_07_08.pdf.

[CIT0018] Guo N., Marra F. & Marra C., 2009, ‘Measuring health-related quality of life in tuberculosis: A systematic review’, *Health and Quality of Life Outcomes* 7(14), 1–10. 10.1186/1477-7525-7-14 19224645 PMC2651863

[CIT0019] Hnizdo E., Singh T. & Churchyard G., 2000, ‘Chronic pulmonary function impairment caused by initial and recurrent pulmonary tuberculosis following treatment’, *Thorax* 55(1), 32–38, viewed n.d., from https://www.ncbi.nlm.nih.gov/pubmed/10607799. 10607799 10.1136/thorax.55.1.32 PMC1745584

[CIT0020] Liistro Vanwelde C., Vincken W., Vandevoorde J., Verleeden G. & Buffels J.G., 2006, ‘Technical and functional assessment of 10 office spirometers: A multicenter a comparative study’, *Chest* 130, 657–665. 10.1378/chest.130.3.657 16963659

[CIT0021] Manie S., Ebrahim T., Miller D., Dreyden T., Simpson R. & Cole G., 2016, ‘Pulmonary impairment after tuberculosis in a South African population’, *South African Journal of Physiotherapy* 72(1), 1–6. 10.4102/sajp.v72i1.307 PMC6093099 30135889

[CIT0022] Marra Marra F., Colley L., Moadebi S., Elwood R. & Fitzgerald J.C., 2008, ‘Health-related quality of life trajectories among adults with tuberculosis – Differences between latent and active infection’, *Chest* 133, 396–403. 10.1378/chest.07-1494 18198260

[CIT0023] Menezes A., Hallal P., Perez-Pidilla R., Jardim J., Muino A., Lopez M. et al., 2007, ‘Tuberculosis and airflow obstruction: Evidence from the PLATINO study in Latin America’, *European Respiratory Journal* 30, 1180–1185. 10.1183/09031936.00083507 17804445

[CIT0024] Mohammad Y., Shaaban R., Abou Al-Zahab B., Khaltaev N., Bousquet J. & Dubaybo B., 2013, ‘Impact of active and passive smoking as risk factors for asthma and COPD in women presenting to primary care in Syria: First report by the WHO-GARD survey group’, *International Journal of Chronic Obstructive Pulmonary Disease* 8, 473–482. 10.2147/Copd.S50551 24124359 PMC3794890

[CIT0025] Plit M.L., Anderson R., Van Rensburg C.E.J., Page-Shipp L., Blott J.A., Fresen J.L. et al., 1998, ‘Influence of antimicrobial chemotherapy on spirometric parameters and pro-inflammatory indices in severe pulmonary tuberculosis’, *European Respiratory Journal* 12(2), 351–356. 10.1183/09031936.98.12020351 9727784

[CIT0026] Pontororing G.J., Kenangalem E., Lolong D.B., Waramori G., Sandjaja, Tjitra E. et al., 2010, ‘The burden and treatment of HIV in tuberculosis patients in Papua Province, Indonesia: A prospective observational study’, *BMC Infectious Diseases* 10(1), 362, 1–9. 10.1186/1471-2334-10-362 21605474 PMC3022835

[CIT0027] Santana M.J.F.D., 2008, *IHE report the importance of measuring health related quality of life*, Institute of Health Economics, Alberta, Canada, viewed 07 July 2019, from https://www.ihe.ca/publications/the-importance-of-measuring-health-related-quality-of-life.

[CIT0028] Sivaranjini S., Vanamail P. & Eason J., 2010, ‘Six minute walk test in people with tuberculosis sequelae’, *Cardiopulmonary Physical Therapy Journal* 21(3), 5–10, viewed n.d., from http://www.ncbi.nlm.nih.gov/pubmed/20957072%0Ahttp://www.pubmedcentral.nih.gov/articlerender.fcgi?artid=PMC2941351. PMC2941351 20957072

[CIT0029] Snider G.L., Doctor L., Demas T.A. & Shaw A.R., 1971, ‘Obstructive airway disease in patients with treated pulmonary tuberculosis’, *American Review of Respiratory Disease* 103(5), 625–640. 10.1164/arrd.1971.103.5.625 5579906

[CIT0030] Ware J., Kosinski M.M. & Keller S., 1996, ‘A 12-item short-form health survey: Construction of scales and preliminary tests of reliability and validity’, *Medical Care* 34(3), 220–233. 10.1097/00005650-199603000-00003 8628042

[CIT0031] World Health Organization, 1964, ‘Preamble to the constitution of the World Health Organization as adopted by the International Health Conference’, New York, 19–22 June 1964, and Entered into Force on 7 April 1948.

[CIT0032] World Health Organization, 2006, *The stop TB strategy, WHO/HTM/TB/2006.368*, viewed 07 July 2019, from http://whqlibdoc.who.int/hq/2006/WHO_HTM_STB_2006.368_eng.pdf.

[CIT0033] World Health Organization, 2015a, *Introducing the end TB strategy*, viewed 07 July 2019, from https://www.who.int/tb/EndTBadvocacy_brochure/en/.

[CIT0034] World Health Organization, 2015b, *WHO global tuberculosis report 2015*, viewed n.d., from http://www.who.int/tb/data.

[CIT0035] Yoshida N., Asai E., Mineta Y., Komatsu Y., Sugiyama Y. & Mineta Y., 2006, ‘Exercise training for the improvement of exercise performance of patients with pulmonary tuberculosis sequelae’, *Internal Medicine* 45(6), 399–403. 10.2169/internalmedicine.45.1505 16617192

